# Cerebrovascular autoregulation and arterial carbon dioxide in patients with acute respiratory distress syndrome: a prospective observational cohort study

**DOI:** 10.1186/s13613-021-00831-7

**Published:** 2021-03-16

**Authors:** Ursula Kahl, Yuanyuan Yu, Axel Nierhaus, Daniel Frings, Barbara Sensen, Anne Daubmann, Stefan Kluge, Marlene Fischer

**Affiliations:** 1 Department of Anesthesiology, University Medical Center Hamburg-Eppendorf, Martinistrasse 52, 20246 Hamburg, Germany; 2 Department of Intensive Care Medicine, University Medical Center Hamburg-Eppendorf, Hamburg, Germany; 3 Institute of Medical Biometry and Epidemiology, University Medical Center Hamburg-Eppendorf, Hamburg, Germany

**Keywords:** Cerebral blood flow, Hypercapnia, Hypocapnia, Respiratory failure, Extracorporeal membrane oxygenation

## Abstract

**Background:**

Early hypercapnia is common in patients with acute respiratory distress syndrome (ARDS) and is associated with increased mortality. Fluctuations of carbon dioxide have been associated with adverse neurological outcome in patients with severe respiratory failure requiring extracorporeal organ support. The aim of this study was to investigate whether early hypercapnia is associated with impaired cerebrovascular autoregulation during the acute phase of ARDS.

**Methods:**

Between December 2018 and November 2019, patients who fulfilled the Berlin criteria for ARDS, were enrolled. Patients with a history of central nervous system disorders, cerebrovascular disease, chronic hypercapnia, or a life expectancy of less than 24 h were excluded from study participation. During the acute phase of ARDS, cerebrovascular autoregulation was measured over two time periods for at least 60 min. Based on the values of mean arterial blood pressure and near-infrared spectroscopy, a cerebral autoregulation index (COx) was calculated. The time with impaired cerebral autoregulation was calculated for each measurement and was compared between patients with and without early hypercapnia [defined as an arterial partial pressure of carbon dioxide (PaCO_2_) ≥ 50 mmHg with a corresponding arterial pH < 7.35 within the first 24 h of ARDS diagnosis].

**Results:**

Of 66 patients included, 117 monitoring episodes were available. The mean age of the study population was 58.5 ± 16 years. 10 patients (15.2%) had mild, 28 (42.4%) moderate, and 28 (42.4%) severe ARDS. Nineteen patients (28.8%) required extracorporeal membrane oxygenation. Early hypercapnia was present in 39 patients (59.1%). Multivariable analysis did not show a significant association between early hypercapnia and impaired cerebrovascular autoregulation (*B* = 0.023 [95% CI − 0.054; 0.100], *p* = 0.556). Hypocapnia during the monitoring period was significantly associated with impaired cerebrovascular autoregulation [*B* = 0.155 (95% CI 0.014; 0.296), *p* = 0.032].

**Conclusion:**

Our results suggest that moderate permissive hypercapnia during the acute phase of ARDS has no adverse effect on cerebrovascular autoregulation and may be tolerated to a certain extent to achieve low tidal volumes. In contrast, episodes of hypocapnia may compromise cerebral blood flow regulation.

*Trial registration* ClinicalTrials.gov; registration number: NCT03949738; date of registration: May 14, 2019

**Supplementary Information:**

The online version contains supplementary material available at 10.1186/s13613-021-00831-7.

## Background

The acute respiratory distress syndrome (ARDS) is a respiratory failure of acute onset, characterized by bilateral pulmonary opacities and severe hypoxemia that cannot be fully explained by cardiac failure or fluid overload [1]. ARDS is common in the intensive care unit (ICU) accounting for about 10% of ICU admissions [2]. Despite advances in the management of ARDS, the mortality remains high with up to 46% in severe ARDS [2]. ARDS survivors suffer from long-term physical disability and cognitive impairment [3].

To allow for low tidal volumes and to avoid ventilator-induced lung injury, the concept of permissive hypercapnia has been proposed in the 1990s [4]. Lower tidal volumes with a moderate hypercapnic acidosis have been shown to improve outcome after ARDS [5]. However, controversy remains on the effects of hypercapnia in ARDS owing to the results of numerous experimental studies suggesting impaired immunological, alveolar epithelial, and hemodynamic function [6–8]. A secondary analysis of three prospective observational trials showed increased ICU mortality in patients with ARDS and early hypercapnia [9]. Aside from hypercapnia, fluctuations of carbon dioxide (CO_2_), have been associated with life-threatening neurological complications in patients requiring extracorporeal membrane oxygenation (ECMO) [10]. Importantly, CO_2_ is one of the most potent vasoactive substances acting on the cerebral circulation, with hypercapnia leading to vasodilation and hypocapnia inducing vasoconstriction [11].

Cerebral blood flow is tightly regulated by autoregulation of the cerebral circulation to ensure a continuous supply of oxygen and nutrients meeting the high cerebral metabolic demand [12]. Cerebrovascular autoregulation (CVA) mediates vasodilation in response to hypotension to avoid cerebral hypoperfusion with the risk of ischemia [13]. In contrast, CVA induces vasoconstriction in response to hypertension to prevent cerebral hyperperfusion [13]. Importantly, there is considerable inter- and intraindividual variation in the upper and lower thresholds of CVA, depending, amongst others, on chronic changes of the cerebral vasculature, the influence of anesthetics, fluctuations in PaCO_2_, and various other mechanisms [13]. Impaired CVA has been observed in critically ill patients and is associated with adverse neurocognitive outcome [14–16].

The aim of this study was to investigate whether early hypercapnia is associated with impaired CVA during the acute phase of ARDS.

## Materials and methods

### Study registration and ethical information

Ethical approval for this study (serial number PV5872) was obtained from the ethics committee of the Hamburg Chamber of Physicians on November 8th, 2018. Oral and written informed consent were obtained from the patient or legal guardian. Details on the informed consent procedure are provided in Additional file 1.

### Design, setting, and participants

This prospective substudy is part of an observational cohort study that follows two aims: (1) to investigate whether early hypercapnia is associated with impaired CVA during the acute phase of ARDS; (2) to assess the association between impaired CVA and self-reported cognitive failures and health-related quality of life. We enrolled patients between December 2018 and November 2019. Adult patients, who were treated for ARDS according to the Berlin definition at the Department of Intensive Care Medicine of the University Medical Center Hamburg-Eppendorf, were screened for eligibility [1]. Patients with a history of central nervous system disorders, cerebrovascular disease, chronic hypercapnia, or a life expectancy of less than 24 h were excluded.

### ARDS management

Patients with moderate or severe hypoxemic respiratory failure refractory to high-flow oxygen or non-invasive ventilation were intubated and mechanically ventilated according to current guidelines and institutional standard operating procedures [17, 18]. Permissive hypercapnia was accepted as long as the arterial pH was higher than 7.2 to ensure lung-protective ventilation with tidal volumes of max. 6 ml/kg of ideal body weight (IBW).

Adjunctive therapies included proning, administration of inhalational nitric oxide, and veno-venous ECMO (vv-ECMO) and were applied according to international and national guidelines [17, 19, 20]. Details on ARDS management throughout the study period are described in Additional file 1.

### Definition of hypercapnia

Early hypercapnia at ARDS onset was defined as arterial partial pressure of CO_2_ (PaCO_2_) ≥ 50 mmHg with a corresponding pH < 7.35 within the first 24 h of ARDS diagnosis. The cut-off for hypercapnia was chosen according to Nin et al., who had shown that a PaCO_2_ ≥ 50 mmHg within the first 48 h of mechanical ventilation was significantly associated with higher mortality [9].

PaCO_2_ during measurement refers to the mean value of two blood gas analyses performed at the beginning and at the end of each CVA monitoring period. PaCO_2_ values were categorized as “normocapnia” (35–50 mmHg), “hypocapnia” (< 35 mmHg), and “hypercapnia” (> 50 mmHg).

### Monitoring of cerebrovascular autoregulation

During the acute phase of ARDS, i.e. within the first 6 days of diagnosis, CVA was measured twice with a minimum interval of 24 h between measurements [21]. Each measurement period had a duration of 60–90 min. CVA was measured during stable respiratory status: one member of the study team supervised the measurement and ensured that ventilation settings (FiO_2_, respiratory rate, inspiratory pressure, positive end-expiratory pressure) were not changed from 30 min before CVA monitoring until measurement completion. If ventilation settings had to be changed for clinical reasons, the measurement was interrupted and resumed later. In patients with vv-ECMO, the CVA measurement was not started until at least 5 h after the beginning of vv-ECMO therapy. For the monitoring of CVA, we used the time correlation method which has been described in detail previously [22–24]. In brief, the cerebral oxygenation index (COx) is calculated from mean arterial pressure (MAP) and cerebral oxygenation (rSO_2_). The MAP was measured continuously with an intra-arterial catheter (Leader-Cath, VYGON GmbH & Co KG, Aachen, Germany) placed in the radial or femoral arteries. Cerebral oxygenation was measured non-invasively with near-infrared spectroscopy (INVOS™ 5100 Cerebral Oximeter, Medtronic, Minneapolis, Minnesota). From the MAP and rSO_2_ values, the COx was depicted as a moving linear correlation coefficient based on a sliding 300-s window that was updated every 10 s (ICM+, Cambridge Enterprise, Cambridge, UK). A COx close to zero indicates intact CVA. In contrast, a positive correlation between rSO_2_ and MAP indicates impaired CVA. COx levels > 0.3 are considered as an indicator of a pathological cerebrovascular autoregulatory response to systemic blood pressure fluctuations [25]. The time with impaired CVA was defined as the percentage of the total monitoring time with a cerebral oxygenation index COx ≥ 0.3.

### Power calculation

We hypothesized that CVA would be more impaired in patients with early hypercapnia compared with patients without early hypercapnia. We aimed to analyze a consecutive sample of patients with ARDS over a 12-month period. Assuming a type I error of 5% (two-tailed hypothesis) and a power of 80%, a sample size of 50 patients would be sufficient to achieve an effect size of 0.81. With a standard deviation of 0.21, a mean difference of 0.17 in COx between groups would be statistically significant. This difference is considered as clinically relevant. We used PASS Version 15.0.3, module “Two-Sample t-Tests using Effect Size” (NCSS, LLC. Kaysville, Utah, USA).

### Data collection

Information on medical history and regular medication was obtained from the patient, next of kin, or the referring physician. Data on critical care management including disease progression, current medication, laboratory parameters, and mechanical ventilation was retrieved from the electronic patient data management system (ICM, Drägerwerk AG & Co. KGaA, Lübeck, Germany) on each day of measurement. The Sequential Organ Failure Assessment score was used to assess sepsis-related multi-organ affection at the time of measurement.

### Statistical analysis

For descriptive statistics, mean and standard deviation or total numbers with percentages were used. Baseline demographic and clinical characteristics were compared between patients with and without early hypercapnia with the Chi-square test, Fisher’s exact test, or Mann–Whitney-*U* test as appropriate. Patients who died before informed consent was obtained were not considered in the statistical analysis. Patients who died before the second monitoring episode were included in the analysis.

For statistical evaluation of association between early hypercapnia and impaired CVA, a linear mixed model fit by restricted maximum likelihood was built including the dependent variable (relative time with impaired CVA), the independent variable of primary interest (early hypercapnia vs. no early hypercapnia) and clinically relevant variables with potential confounding influence (age, Sequential Organ Failure Assessment score, ARDS severity, ARDS etiology, sedation, PaCO_2_ during CVA assessment, prone position, inhalational nitric oxide, vv-ECMO) as fixed effects and patient as a random effect. The model was gradually reduced following a stepwise-backwards approach. Variables that caused a change in parameter estimates of > 10% or that were statistically significant (*p* < 0.05, two-tailed hypotheses) remained in the model. The linearity between continuous variables was checked graphically with scatter plots. Residuals were graphically assessed for normal distribution using Q–Q and residual plots. Additionally, we performed a leave-one-out cross-validation to evaluate the final model. As part of a sensitivity analysis, the model was re-calculated with (1) a higher threshold for early hypercapnia (PaCO_2_ ≥ 60 mmHg with a corresponding pH < 7.35 within the first 24 h of ARDS diagnosis) and (2) delta PaCO_2_ (the difference between ARDS diagnosis and CVA assessment).

An exploratory subgroup analysis was performed to compare CVA during measurements with and without vv-ECMO. In patients, who received vv-ECMO between two measurements, CVA was compared before and after vv-ECMO initiation.

All statistical analyses were performed with SPSS Version 24 (IBM SPSS Statistics, IBM Corporation). Figures were designed with Prism 8, Version 8.4.3 (GraphPad Software Inc., San Diego, CA, USA).

## Results

### Patient characteristics

**Fig. 1:**
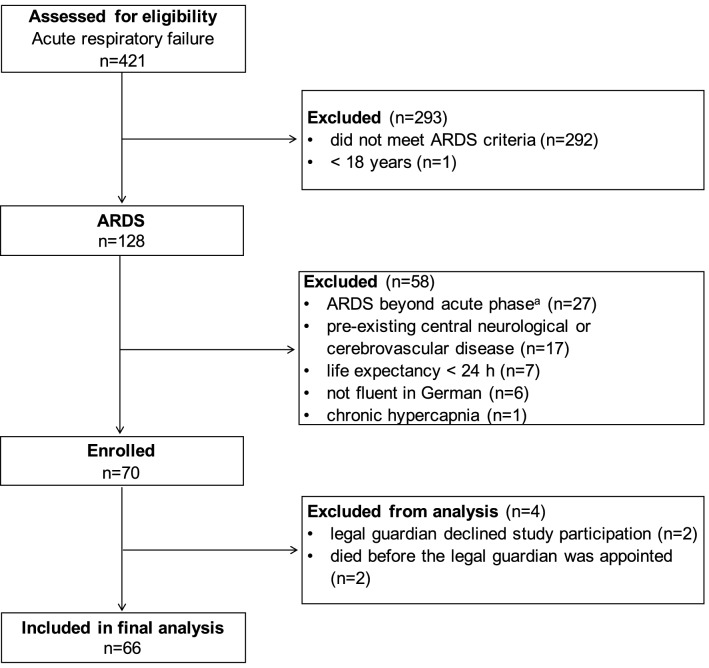
Flowchart of patient selection and enrolment. ^a^> 6 days from the onset of acute respiratory distress syndrome (ARDS)

Between December 2018 and November 2019, 70 patients were enrolled and 66 patients were included in the final analysis. Data from 2 patients, who died before a legal guardian was appointed or before they regained consciousness and ability to give informed consent, were destroyed and therefore not available for analysis. Legal guardians from two patients did not give consent to study participation. Data from the remaining patients were collected completely, so there are no missing values in our dataset. Figure 1 shows the flow of participants throughout the study.

The mean age of the study population was 58.5 years (± 16); 14 patients (21.2%) were female. The majority of patients fulfilled the criteria for moderate (*n* = 28, 42.4%) or severe (*n* = 28; 42.4%) ARDS. PaO_2_/FiO_2_ ratios and PEEP values for individual study participants are presented in Additional file 2. Etiologies of ARDS included pneumonia (*n* = 45, 68.2%), extrapulmonary causes (*n* = 12, 18.2%), toxic lung injury (*n* = 3, 4.5%), aspiration of gastric contents (*n* = 3; 4.5%) and unknown causes (*n* = 3, 4.5%). Early hypercapnia was present in 39 of 66 patients (59.1%). All patients required mechanical ventilation. Seven patients (10.6%) were ventilated non-invasively. A total of 19 patients (28.8%) received vv-ECMO. Baseline demographics, clinical characteristics and details on ARDS management stratified by the presence of early hypercapnia are presented in Table 1. Data on new central nervous system disorders at discharge and functional outcome at 3 months are presented in Additional file 3.

**Table 1:** Baseline demographic and clinical characteristics and details on the management of acute respiratory distress syndrome (ARDS)

	No early hypercapnia (*n* = 27)	Early hypercapnia (*n* = 39)	*p*
Age, years	62 ± 17	55 ± 15	0.040
Gender (female)	5 (18.5)	9 (23.1)	0.765
Body mass index	26.8 ± 4.4	30.8 ± 11.3	0.334
Comorbid conditions			
Arterial hypertension	10 (37.0)	16 (41.0)	0.802
Diabetes	1 (3.7)	8 (20.5)	0.071
Coronary heart disease	3 (11.1)	7 (17.9)	0.508
Arrhythmia	7 (25.9)	3 (7.7)	0.077
Chronic obstructive pulmonary disease^a^	2 (7.4)	6 (15.4)	0.455
Asthma	0 (0.0)	2 (5.1)	0.509
Malignant hematooncologic disease	7 (25.9)	3 (7.7)	0.077
Autoimmune disease	3 (11.1)	2 (5.1)	0.393
Solid organ transplantation	2 (7.4)	2 (5.1)	1.000
AIDS	0 (0.0)	1 (2.6)	1.000
ARDS severity			0.397
Mild	4 (14.8)	6 (15.4)	
Moderate	14 (51.9)	14 (35.9)	
Severe	9 (33.3)	19 (48.7)	
ARDS etiology			0.136
Community-acquired pneumonia	9 (33.3)	19 (48.7)	
Hospital-acquired pneumonia	11 (40.7)	6 (15.4)	
Aspiration	1 (3.7)	2 (5.1)	
Toxic	2 (7.4)	1 (2.6)	
Extrapulmonary	4 (14.8)	8 (20.5)	
Unknown	0 (0.0)	3 (7.7)	
ARDS etiology			0.020
Community-acquired	12 (44.4)	29 (74.4)	
Hospital-acquired	15 (55.5)	10 (25.6)	
SOFA score^b^	9 ± 4	10 ± 3	0.854
Mechanical ventilation			
Non-invasive ventilation	7 (25.9)	0 (0.0)	< 0.001
Invasive ventilation	20 (74.1)	39 (100)	
Respiratory rate, breaths per min	25 ± 5	21 ± 6	< 0.001
Tidal volume, ml	434 ± 185	395 ± 133	0.485
Difference from ideal tidal volume^c^, ml	19 ± 192	− 36 ± 142	0.264
Positive end-expiratory pressure, mbar	10 ± 4	13 ± 4	0.001
Driving pressure, mbar	15 ± 4	14 ± 3	0.231
Sedation			0.048
Intravenous^d^	13 (48.1)	17 (43.6)	
Inhalational^e^	0 (0.0)	2 (5.1)	
Mixed^f^	10 (37.0)	20 (51.3)	
None	4 (14.8)	0 (0.0)	
Adjunctive therapy			
Prone position	11 (40.7)	14 (35.9)	0.798
Inhaled nitric oxide	6 (22.2)	9 (23.1)	1.000
Veno-venous ECMO	3 (11.1)	16 (41.0)	0.012

Data are given in *n* (%) or mean ± SD

*ECMO* extracorporeal membrane oxygenation

^a^Without chronic hypercapnia

^b^Highest score during measurement of cerebrovascular autoregulation

^c^Ideal tidal volume was defined as 6 ml/kg of ideal body weight

^d^Continuous administration of propofol or midazolam

^e^Inhalational application of isoflurane

^f^Propofol plus isoflurane or midazolam plus isoflurane

### Early hypercapnia

**Fig. 2:**
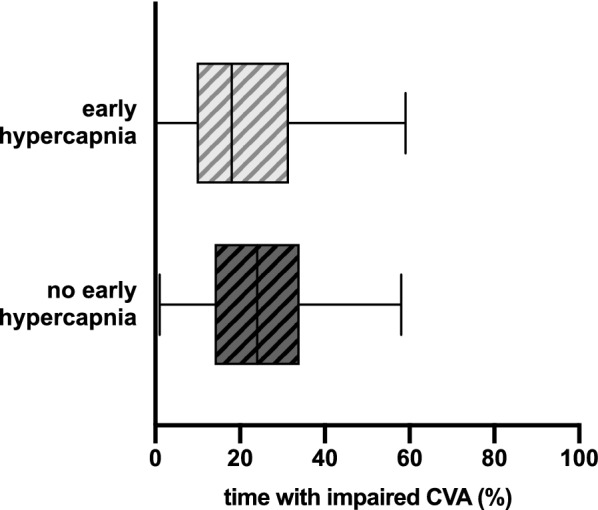
Early hypercapnia and cerebrovascular autoregulation. Shows the time with impaired cerebrovascular autoregulation (CVA; in % of the total monitoring time) in ARDS patients with early hypercapnia vs. no early hypercapnia. The COx was depicted as a moving linear correlation based on mean arterial pressure and cerebral oxygenation (ICM+, Cambridge Enterprise, Cambridge, UK). Impaired cerebrovascular autoregulation was defined as a COx > 0.3. *ARDS* Acute respiratory distress syndrome, *COx* cerebral oxygenation index. Data are presented as median (boxes) with Tukey whiskers

Cerebrovascular autoregulation was monitored twice in 51 of the 66 patients, and once in 15 patients, resulting in a total of 117 measurement periods. Patients with early hypercapnia showed impaired CVA during 22.95 ± 18.75% of the monitoring period. In patients without early hypercapnia cerebrovascular autoregulatory response was impaired during 27.45 ± 18.51% of the monitoring period (Fig. 2). Mean MAP (74.9 mmHg ± 9.1 vs. 76.3 mmHg ± 10.8) and mean COx (0.06 ± 0.18 vs. 0.11 ± 0.15) were similar in patients with and without early hypercapnia. Cerebral oxygenation was higher in patients with early hypercapnia (67.32% ± 10.20) compared with patients without early hypercapnia (59.81% ± 12.03). Patients with early hypercapnia had significantly higher mean PaCO_2_ (47.8 mmHg ± 17.9 vs. 41.6 mmHg ± 8.6, *p* = 0.001) and higher PaCO_2_ variability (115.1 mmHg ± 168.8 vs. 32.1 mmHg ± 40.3, *p* < 0.001) between ARDS diagnosis and the first CVA assessment compared with patients without early hypercapnia; Table 2. The evolution of PaCO_2_ between ARDS onset and CVA assessments is presented in Additional file 4. Additional data on hemodynamic parameters and results from arterial blood gas analyses are listed in Table 2.

**Table 2:** Hemodynamic parameters and selected results from blood gas analyses during the measurement of cerebrovascular autoregulation (CVA)

	No early hypercapnia (*n* = 27)	Early hypercapnia (*n* = 39)
	Measurement periods = 47	Measurement periods = 70
Time with impaired CVA (%)	27.45 ± 18.51	22.95 ± 18.75
Duration of measurement (min)	77 ± 12	77 ± 15
Time with impaired CVA (min)	21 ± 15	18 ± 15
Cerebral oxygenation index COx	0.11 ± 0.15	0.06 ± 0.18
Regional cerebral oxygen saturation rSO_2_ (%)	59.81 ± 12.03	67.32 ± 10.20
Mean arterial blood pressure (mmHg)	76.34 ± 10.84	74.85 ± 9.12
Heart rate (bpm)	81.25 ± 18.80	90.45 ± 19.10
Body temperature (°C)	37.1 ± 0.8	37.1 ± 0.9
Hemoglobin (mg dl^−1^)	9.20 ± 1.42	9.79 ± 1.63
PaO_2_/FiO_2_ ratio	152.55 ± 57.16	151.38 ± 75.27
Mean PaO_2_ (mmHg)	80.6 ± 18.4	85.9 ± 16.4
Mean PaCO_2_ (mmHg)^a^	45.2 ± 11.1	47.7 ± 9.6
Hypocapnia (< 35 mmHg)^b^	8 (17)	3 (4.3)
Normocapnia (35–50 mmHg)^b^	28 (59.6)	43 (61.4)
Hypercapnia (> 50 mmHg)^b^	11 (23.4)	24 (34.3)
pH	7.41 ± 0.08	7.39 ± 0.09
Lactate (mmol/L)	1.6 ± 0.9	2.3 ± 2.5
Δ PaCO_2_ (mmHg)^c^	1.29 ± 1.76	1.79 ± 3.23
Δ pH^c^	0.01 ± 0.02	0.01 ± 0.02
Δ PaO_2_/FiO_2_ ratio (mmHg)^c^	8.20 ± 12.86	9.86 ± 19.73
Isoflurane (ml/h)	6.5 ± 4.5	6.8 ± 4.1
Isoflurane (end-tidal concentration in %)	1.1 ± 0.4	1.2 ± 0.3
From ARDS diagnosis until first CVA measurement		
Time from ARDS diagnosis to first measurement (days)	4 ± 4	2 ± 2
Mean PaCO_2_ (mmHg)	41.6 ± 8.6	47.8 ± 17.9
PaCO_2_ variability^d^ (mmHg)	32.1 ± 40.3	115.1 ± 168.8
From first until second CVA measurement		
Time from ARDS diagnosis to second measurement (days)	8 ± 3	5 ± 3
Mean PaCO_2_ (mmHg)	49.2 ± 10.9	49.0 ± 7.3
PaCO_2_ variability^d^ (mmHg)	68.6 ± 65.2	57.8 ± 50.2

Mean values from monitoring episodes 1 and 2, stratified by the presence of early hypercapnia. Data are given in *n* (%) or mean ± SD

*COx* cerebral oxygenation index as a surrogate of cerebrovascular autoregulation

^a^“Mean PaCO_2_” refers to the statistical mean of two blood gas analyses during one CVA monitoring episode

^b^PaCO_2_ was categorized as “hypocapnia”, “normocapnia”, and “hypercapnia” during each measurement period

^c^Δ values refer to the mean difference between the first and the second CVA measurement in one study participant

^d^Calculated as the variance

**Fig. 3:**
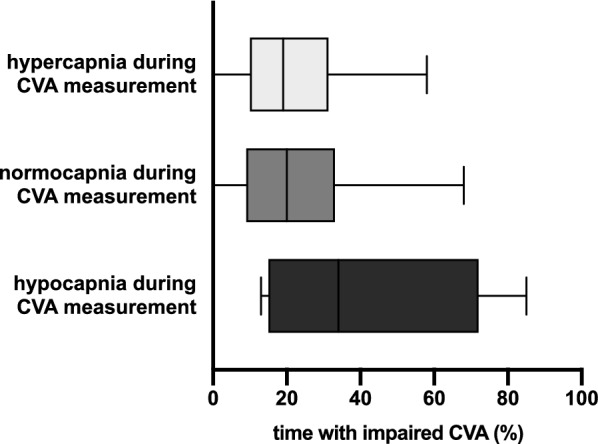
Cerebrovascular autoregulation and carbon dioxide during measurement periods. The time with impaired cerebrovascular autoregulation (CVA, in % of the total monitoring time) is shown for patients with hypocapnia (PaCO_2_ < 35 mmHg), normocapnia (PaCO_2_ 35–50 mmHg) and hypercapnia (PaCO_2_ > 50 mmHg) during the measurement of CVA. Data are presented as median (boxes) with Tukey whiskers

Multivariable analysis did not show a significant association between early hypercapnia (PaCO_2_ ≥ 50 mmHg with pH < 7.35) and impaired CVA (Table 3). In contrast to normo- and hypercapnia, hypocapnic episodes during measurement were significantly associated with impaired CVA (*B* = 0.155 [95% CI 0.014; 0.296], *p* = 0.032); Fig. 3. Other variables included in the model for a potentially confounding influence (vv-ECMO, sedation, ARDS severity, age, ARDS etiology) were not significantly associated with impaired CVA (Table 3 and Additional file 5).

**Table 3:** Linear mixed model—estimates of fixed effects

Parameter	Estimate	95% CI—low	95% CI—up	*p*
Intercept	0.177	− 0.059	0.414	0.139
No early hypercapnia (vs. early hypercapnia = PaCO_2_ ≥ 50 mmHg)	0.023	− 0.054	0.100	0.556
Sedation				
Mixed sedation (vs. no sedation)	− 0.042	− 0.217	0.133	0.635
Intravenous sedation (vs. no sedation)	− 0.074	− 0.235	0.087	0.363
Inhalational sedation (vs. no sedation)	− 0.076	− 0.260	0.107	0.410
ARDS severity				
Mild (vs. severe)	0.032	− 0.062	0.126	0.498
Moderate (vs. severe)	− 0.018	− 0.102	0.065	0.663
Age (per year increase)	0.001	− 0.001	0.004	0.275
Hypocapnia during the measurement period^a^	0.155	0.014	0.296	0.032
ARDS etiology (community-acquired vs. hospital-acquired)^b^	0.047	− 0.027	0.122	0.208

The variables position (prone vs. supine), inhalational nitric oxide, extracorporeal membrane oxygenation, and the Sequential Organ Failure Assessment score during measurement were included in the initial model and eliminated during the stepwise-backwards reduction

ARDS: acute respiratory distress syndrome

^a^Vs. Normo- and hypercapnia

^b^Etiologies were categorized as “community-acquired” and “hospital-acquired” for the linear mixed model

The sensitivity analysis did not show a significant association between early hypercapnia (PaCO_2_ ≥ 60 mmHg) and impaired CVA (Additional file 6a). In contrast to normo- and hypercapnia, hypocapnic episodes during measurement were significantly associated with impaired CVA (*B* = 0.186 [95% CI 0.072; 0.300], *p* = 0.002). Other variables included in the model for a potentially confounding influence (age and ARDS etiology) were not significantly associated with impaired CVA (Additional file 6a).

To assess the potential effect of PaCO_2_ changes on CVA impairment, we used another linear mixed model with the difference in PaCO_2_ between ARDS diagnosis and the time of CVA assessment (Additional file 6b). There was no significant association of the delta PaCO_2_ with impaired CVA. Compared with normo- and hypercapnia, hypocapnia during CVA measurement was significantly associated with impaired CVA.

### Extracorporeal membrane oxygenation

Cerebrovascular autoregulation was compared between 19 patients with vv-ECMO, and 47 patients without vv-ECMO. Mean COx and the time with impaired CVA were similar between vv-ECMO and non-ECMO patients (Additional file 7). Patients with vv-ECMO had higher cerebral oxygenation (rSO_2_ = 67.32% ± 10.96) than patients without vv-ECMO (rSO_2_ = 63.21% ± 11.6). In seven patients, who received vv-ECMO during the course of the disease, CVA was measured before and after vv-ECMO initiation. Results from these paired monitoring episodes are shown in Additional file 8.

## Discussion

The aim of this prospective observational study was to compare CVA during the acute phase of ARDS between patients with and without early hypercapnia. We found that early hypercapnia was not associated with impaired CVA. However, we observed a significant association between hypocapnia during the acute phase of ARDS and impaired CVA.

Early hypercapnia has been shown to be associated with adverse outcome after ARDS [8, 9]. Previous clinical trials that investigated CVA in ARDS focused primarily on the influence of PEEP rather than CO_2_ [26, 27]. While Yang et al. did not specifically address the cerebrovascular effects of PaCO_2_, Schramm et al. reported that hypercapnia was not associated with impaired CVA, which is similar to our results.

We found that hypocapnia during measurement was associated with impaired CVA. Interestingly, in a large retrospective registry analysis, Cavayas et al. found the rate of PaCO_2_ reduction upon ECMO initiation to be associated with neurological complications in patients with severe respiratory failure [10]. The risk for ischemic stroke, intracerebral hemorrhage, seizures, and brain death increased with the magnitude of PaCO_2_ correction within 24 h after ECMO initiation [10]. Similar results were reported in a retrospective single-center study [28]. In 135 patients on vv-ECMO for severe respiratory failure, rapid correction of hypercapnia was associated with intracranial bleeding [28]. We did not find a significant association of the difference in PaCO_2_ between ARDS diagnosis and CVA assessment and impaired CVA. However, we did not analyze the effect of hypercapnia reduction on CVA.

There are conflicting results on the effect of hypocapnia on CVA in pathologies other than ARDS. While hypocapnia has been shown to promote the impairment of CVA in patients with acute brain injury [29], other studies found no adverse effect of transient hypocapnia on CVA in healthy adults and other neurocritical care patients [30–33].

When interpreting the conflicting results on the effects of CO_2_ on CVA, it is important to differentiate between the diverse patient populations, as well as different definitions of hypo-, normo- and hypercapnia. This may limit the generalizability of results from single trials. Furthermore, the duration of hyper- and hypocapnia has to be considered when analyzing potential effects of CO_2_ on CVA. In patients with ARDS, alterations of PaCO_2_ are mainly driven by respiratory failure or ventilation strategies including low tidal volumes and permissive hypercapnia. Due to our strictly observational study design, we did not actively change PaCO_2_. Therefore, hyper- or hypocapnia had been present for a period of several hours up to 6 days before measurement. In contrast, studies in healthy subjects and neurocritical care patients included interventional modifications of ventilator settings or spontaneous inhalation of CO_2_ [29, 31, 33]. In these trials, there was little time delay between reaching the target PaCO_2_ and the measurement of CVA. Muizelaar et al. observed that the vasoconstrictive effect of hyperventilation decreases over time. They showed that cerebral vessels returned to their baseline diameter after 20 h of hyperventilation [34]. One can assume that the effects of CO_2_ on vascular tone become apparent early after elevation or reduction of PaCO_2_ and that the cerebral vasculature adapts to intermediate or long-term alterations of PaCO_2_.

In our study, almost 60% of patients presented with early hypercapnia, which is higher than reported previously [9]. Notably, Nin et al. found a 22% prevalence of early hypercapnia in patients with ARDS [9]. The different prevalence may be caused by two main factors: we defined early hypercapnia within the first 24 h of ARDS diagnosis, while Nin et al. assessed hypercapnia during the first 48 h of mechanical ventilation [9]. Importantly, we used protective ventilation with tidal volumes of ≤ 6 ml/kg of IBW. In contrast, Nin et al. performed a secondary analysis of data from three clinical trials that were published between 2002 and 2013 [35–37]. Mean tidal volumes in these trials were substantially higher with up to 10 ml/kg of IBW, probably reflecting the gradual implementation of the concept of low tidal volumes and permissive hypercapnia into clinical practice.

We observed higher cerebral oxygenation in patients with early hypercapnia compared to patients without early hypercapnia. This finding might be attributable to the vasodilatory effect of CO_2_ leading to an increase in cerebral blood flow and cerebral tissue oxygenation [11]. Of note, patients with early hypercapnia received sedation with isoflurane more frequently. Volatile anesthetics such as isoflurane are characterized by cerebral vasodilatory properties that may contribute to higher cerebral oxygenation in patients with early hypercapnia [38].

Our study was primarily designed to assess the effect of early hypercapnia on CVA in patients with ARDS. It is important to note that CO_2_ does not only influence cerebral vascular tone, but also other organ systems. Among others, hyper- and hypocapnia are associated with the severity of respiratory failure, compromised immune response, and impaired right ventricular function [8, 39].

### Limitations and strengths

There are several limitations to this observational trial. First, spontaneously breathing patients without sedation were more likely to have higher respiratory rates and lower PaCO_2_. We did consider the type of sedation as a potential confounder in the multivariable analysis and did not find a significant association between type of sedation and CVA. However, anesthetics are known to affect cerebrovascular tone as well as CVA. We cannot rule out that a statistical effect did not become apparent because of our relatively small sample size. The same applies for vv-ECMO therapy, which was not significantly associated with impaired CVA in our study. Importantly, the impact of vv-ECMO on CVA remains unclear [40]. Having said that, it is noteworthy that we used veno-venous flow in all patients requiring ECMO, which is supposed to have less impact on cerebral blood flow than ECMO with veno-arterial flow [41].

Second, there are several methods for the continuous measurement of CVA that differ with regard to the assessment of cerebral blood flow. Non-invasive approaches include transcranial Doppler sonography and near-infrared spectroscopy [24]. We chose the latter approach, which has been validated thoroughly [42, 43]. However, differences in methodological approaches lead to a limited comparability between study results. We continuously analyzed CVA during a 60–90 min period during the acute phase of ARDS. PaCO_2_ was determined twice during CVA assessment. There were no substantial fluctuations of PaCO_2_ during CVA monitoring. However, we observed a high variability of PaCO_2_ between the diagnosis of ARDS and CVA assessment that was significantly higher in patients with early hypercapnia. By choosing CVA assessment within predefined time periods of 60–90 min, we may have missed relevant fluctuations of PaCO_2._ This limitation highlights the need for future studies using continuous CVA monitoring throughout the course of disease.

Two-channel near-infrared spectroscopy used in this study provides information on cerebral oxygenation in the area of the frontal cortex. Oxygenation of other cerebral lobes and deeper brain structures is not reflected by near-infrared spectroscopy [44].

Third, volatile anesthetics including isoflurane may have a profound effect on cerebrovascular tone resulting in a substantial impact on CVA [45]. Although we have included the type of sedation as a potential confounder in the multivariable analysis, the cerebrovascular effects of isoflurane may have biased the results of our study.

Since the definition of hypercapnia varies between studies, we performed a sensitivity analysis using a higher threshold for early hypercapnia (PaCO_2_ ≥ 60 mmHg). This confirmed our initial findings and did not reveal an association between early hypercapnia and impaired CVA. Impairment of CVA has been linked with poor neurocognitive outcome and mortality in patients without structural central nervous system lesions [14, 43]. This study focused on the association between alterations of PaCO_2_ during the acute phase of ARDS and CVA. Future research should assess the effect on CVA impairment on functional neurological outcome after ARDS.

## Conclusions

In the present study, we did not observe an adverse impact of hypercapnia during the acute phase of ARDS on CVA. However, we found that hypocapnia is associated with impaired CVA. Our findings suggest that moderate hypercapnia during the acute phase of ARDS may be safe with regard to CVA and hypercapnia may be tolerated to a certain extent to achieve low tidal volumes, whereas episodes of hypocapnia may compromise cerebral blood flow regulation.

## Supplementary Information

**Additional file 1.** ARDS management. Informed consent procedure, ARDS diagnosis, and ARDS management throughout the study period.

**Additional file 2.** PaO_2_/FiO_2_ ratio and PEEP for ARDS diagnosis. PaO_2_/FiO_2_ ratio and positive end-exspiratory pressure (PEEP) in individual study participants.

**Additional file 3.** CVA and functional outcome. Neuroimaging, new CNS disorders, and functional outcome at three months.

**Additional file 4.** Development of arterial carbon dioxide. PaCO2 from the diagnosis of ARDS until first CVA assessment and between first and second CVA assessments, stratified by the presence of early hypercapnia.

**Additional file 5.** Linear mixed model including preselected variables of clinical relevance. Initial linear mixed model. All variables included were selected based on clinical considerations.

**Additional file 6.** Sensitivity analysis. 6a—Linear mixed model with an alternative definition of early hypercapnia (PaCO_2_ ≥ 60 mmHg with pH < 7.35). 6b—Linear mixed model with delta PaCO2 (difference between ARDS diagnosis and CVA measurements) as independent variable.

**Additional file 7.** CVA in patients with and without ECMO. 7a—Hemodynamic parameters and selected results from blood gas analyses in patients requiring extracorporeal membrane oxygenation (ECMO) and patients without ECMO. Mean values from monitoring episodes 1 and 2 are presented, stratified by the requirement of veno-venous ECMO. Data are given as mean ± SD. COx: cerebral oxygenation index representing cerebrovascular autoregulation (CVA). rSO_2_: cerebral oxygenation measured with near-infrared spectroscopy. MAP: mean arterial blood pressure. 7b—Time with impaired CVA between 19 patients with veno-venous ECMO, and 47 patients without ECMO.

**Additional file 8.** Repeated CVA measurements in 7 patients with and without ECMO. 8a—Repeated measurement episodes of cerebrovascular autoregulation (CVA) with and without extracorporeal membrane oxygenation (ECMO) in 7 patients. Data are given as mean ± SD. COx: cerebral oxygenation index as a surrogate of CVA. rSO_2_: cerebral oxygenation measured with near-infrared spectroscopy. 8b—Time with impaired CVA in repeated measurements with and without ECMO.

## Data Availability

The datasets used and analyzed during the current study are available from the corresponding author on reasonable request.

## Abbreviations

ARDS: Acute respiratory distress syndrome
CO_2_: Carbon dioxide
COx: Cerebral oxygenation index
CVA: Cerebrovascular autoregulation
vv-ECMO: Veno-venous extracorporeal membrane oxygenation
IBW: Ideal body weight
MAP: Mean arterial blood pressure
PaCO_2_: Arterial partial pressure of carbon dioxide
