# Supplementary material for: Cerebrovascular autoregulation and arterial carbon dioxide in patients with acute respiratory distress syndrome: a prospective observational cohort study
Source: Ann Intensive Care. 2021 Mar 16;11:47. doi: 10.1186/s13613-021-00831-7 (PMC7962086; doi:10.1186/s13613-021-00831-7)
Supplement: Supplementary file 1 — Additional file 1. ARDS management. Informed consent procedure, ARDS diagnosis, and ARDS management throughout the study period. [file 13613_2021_831_MOESM1_ESM.docx]

**Additional file 1**

*Informed consent*

Informed consent was obtained in accordance with German law and the regulations of the Hamburg Chamber of Physicians’ ethics committee.

• Scenario 1: Oral and written informed consent were obtained from the patient or legal guardian before enrolment.

• Scenario 2: For eligible patients, who were unable to give consent and did not have a legal guardian, the following proceedings applied: Based on the presumed patient's will that was determined with the next of kin and after consultation with an independent physician, who was not involved in critical care management, the preliminary decision to participate in the study or decline to study participation was made. Written consent was obtained as soon as a legal guardian had been appointed or the patient regained consciousness.

*ARDS diagnosis*

As part of the routine work-up in the diagnosis of ARDS, all patients receive an x-ray or a computed tomography scan of the chest. To rule out a cardiac etiology of acute respiratory failure, a transthoracic echocardiography is performed in all patients.

*ARDS management*

Patients with beginning respiratory failure received high-flow nasal oxygen therapy or non-invasive ventilation. Patients with moderate to severe hypoxemic respiratory failure refractory to high-flow oxygen or non-invasive ventilation were intubated. Mechanical ventilation was provided according to current guidelines and institutional standard operating procedures [1, 2]. Permissive hypercapnia was tolerated as long as the arterial pH was higher than 7.2 to ensure lung-protective ventilation with tidal volumes of max. 6 ml/kg ideal body weight.

Patients, who were invasively ventilated and had an FiO_2_/PaO_2_ ratio < 150 mmHg were continuously proned for at least 16 hours. In patients with acute severe hypoxemia (requiring FiO_2_ of 0.8 or more) or with right heart failure, inhaled nitric oxide was applied for pulmonary vasodilation. As a final escalation strategy, patients with severe hypoxemic and/or hypercapnic respiratory failure in combination with severe respiratory acidosis refractory to adjunctive therapies received veno-venous extracorporeal membrane oxygenation (ECMO; CARDIOHELP-System Maquet GmbH, Rastatt, Germany or Novalung, Fresenius Medical Care, Bad Homburg, Germany).

For circulatory support, patients were treated with restrictive fluid management after initial resuscitation and continuous norepinephrine to maintain a mean arterial pressure of ≥65 mmHg. If required, sedation was titrated to achieve a score of 0 or -1 on the Richmond Agitation and Sedation Scale (RASS), which ranges from -5 (unarousable) to +4 (combative). In patients who required neuromuscular blockade to facilitate mechanical ventilation and to reduce patient-ventilator dyssynchrony or in patients with severe hemodynamic instability, lower RASS scores were aimed at. For sedation, propofol, midazolam, isoflurane, or a combination of these substances were used. Pain management was performed according to current guidelines using opioid-based analgesia [3]. If signs of infection were present, patients received calculated or specific anti-infective medication according to suspected or detected pathogens.

**References**

1. Fan E, Del Sorbo L, Goligher EC, et al (2017) An Official American Thoracic Society/European Society of Intensive Care Medicine/Society of Critical Care Medicine Clinical Practice Guideline: Mechanical Ventilation in Adult Patients with Acute Respiratory Distress Syndrome. Am J Respir Crit Care Med 195:1253–1263

2. Fichtner F, Moerer O, Weber-Carstens S, Nothacker M, Kaisers U, Laudi S, Guideline group (2019) Clinical Guideline for Treating Acute Respiratory Insufficiency with Invasive Ventilation and Extracorporeal Membrane Oxygenation: Evidence-Based Recommendations for Choosing Modes and Setting Parameters of Mechanical Ventilation. Respir Int Rev Thorac Dis 98:357–372

3. DAS-Taskforce 2015, Baron R, Binder A, et al (2015) Evidence and consensus based guideline for the management of delirium, analgesia, and sedation in intensive care medicine. Revision 2015 (DAS-Guideline 2015) - short version. Ger Med Sci GMS E-J 13:Doc19
