# Supplementary material for: Cerebrovascular autoregulation and arterial carbon dioxide in patients with acute respiratory distress syndrome: a prospective observational cohort study
Source: Ann Intensive Care. 2021 Mar 16;11:47. doi: 10.1186/s13613-021-00831-7 (PMC7962086; doi:10.1186/s13613-021-00831-7)
Supplement: Supplementary file 2 — Additional file 2. PaO2/FiO2 ratio and PEEP for ARDS diagnosis. PaO2/FiO2 ratio and positive end-exspiratory pressure (PEEP) in individual study participants. [file 13613_2021_831_MOESM2_ESM.docx]

**Additional file 2**

| Patient | PaO_2_/FiO_2_ ratio (mmHg) | PEEP (mbar) |
| --- | --- | --- |
| 1 | 170 | 15 |
| 2 | 41.7 | 13 |
| 3 | 108 | 16 |
| 4 | 130 | 12 |
| 5 | 94.9 | 8 |
| 6 | 92.8 | 5 |
| 7 | 68.2 | 5 |
| 8 | 267 | 5 |
| 9 | 133 | 5 |
| 10 | 104 | 5 |
| 11 | 97.1 | 13 |
| 12 | 179 | 5.2 |
| 13 | 93.3 | 8 |
| 14 | 131 | 12 |
| 15 | 132 | 15 |
| 16 | 133 | 5 |
| 17 | 184 | 8 |
| 18 | 48.6 | 14 |
| 19 | 77.6 | 13 |
| 20 | 58 | 12 |
| 21 | 53.4 | 16 |
| 22 | 109 | 6.2 |
| 23 | 70.2 | 15 |
| 24 | 182 | 13 |
| 25 | 230 | 10 |
| 26 | 118 | 10 |
| 27 | 169 | 8 |
| 28 | 205 | 12 |
| 29 | 190 | 5 |
| 30 | 235 | 12 |
| 31 | 99.6 | 15 |
| 32 | 197 | 8 |
| 33 | 139 | 20 |
| 34 | 98.8 | 9 |
| 35 | 73.3 | 9 |
| 36 | 58.4 | 16 |
| 37 | 101 | 8 |
| 38 | 71.4 | 7.3 |
| 39 | 139 | 5.2 |
| 40 | 118 | 12 |
| 41 | 61.1 | 5 |
| 42 | 180 | 6 |
| 43 | 104 | 8.4 |
| 44 | 75.2 | 7.8 |
| 45 | 54 | 15 |
| 46 | 171 | 6.4 |
| 47 | 184 | 10 |
| 48 | 186 | 7 |
| 49 | 134 | 8.5 |
| 50 | 230 | 9.1 |
| 51 | 111 | 5 |
| 52 | 74.6 | 5 |
| 53 | 104 | 14 |
| 54 | 102 | 14 |
| 55 | 103 | 5 |
| 56 | 72.7 | 5 |
| 57 | 158 | 7 |
| 58 | 114 | 15 |
| 59 | 128 | 5 |
| 60 | 128 | 5 |
| 61 | 102 | 5 |
| 62 | 164 | 5 |
| 63 | 60.3 | 14 |
| 64 | 110 | 13 |
| 65 | 114 | 9 |
| 66 | 97.2 | 11 |

**Additional file 2**: PaO_2_/FiO_2_ ratio and positive end-exspiratory pressure (PEEP) in individual study participants.
