# Supplementary material for: Cerebrovascular autoregulation and arterial carbon dioxide in patients with acute respiratory distress syndrome: a prospective observational cohort study
Source: Ann Intensive Care. 2021 Mar 16;11:47. doi: 10.1186/s13613-021-00831-7 (PMC7962086; doi:10.1186/s13613-021-00831-7)
Supplement: Supplementary file 3 — Additional file 3. CVA and functional outcome. Neuroimaging, new CNS disorders, and functional outcome at three months. [file 13613_2021_831_MOESM3_ESM.docx]

**Additional file 3**

|  | n (%) | % of time with impaired CVA |
| --- | --- | --- |
| *Acute brain injury during the ICU stay^a^* | | |
| *No cerebral CT scan* | 33 (50) | 21.9 ± 16.9 |
| *Cerebral CT without new ABI* | 23 (34.8) | 28.1 ± 13.7 |
| *Intracranial hemorrhage* | 4 (6.1) | 25.8 ± 11.6 |
| *Cerebral ischemia* | 2 (3) | 22.8 ± 7.1 |
| *Other^b^* | 4 (6.1) | 23.2 ± 7.9 |
| *New central nervous system disorder^c^* | | |
| *No focal neurological symptoms* | 29 (43.9) | 25.4 ± 19 |
| *Encephalopathy* | 3 (4.5) | 21.4 ± 0.4 |
| *Seizure* | 3 (4.5) | 31.2 ± 3.9 |
| *Non-survivor* | 31 (47) | 23 ± 11.7 |
| *Functional outcome at 3 months^d^* | |  |
| *Full recovery or mild disability* | 10 (15.2) | 23.6 ± 17.5 |
| *Moderate disability but independence in activities of daily living* | 12 (18.2) | 35.2 ± 21.1 |
| *Severe disability; dependent in activities of daily living* | 9 (13.6) | 17.7 ± 9.7 |
| *Coma or persistent vegetive state* | 0 (0) |  |
| *Dead* | 33 (50) | 22.9 ± 11.3 |

**Additional file 3**. Mean percentage of time with impaired cerebrovascular autoregulation (CVA), stratified by (a) acute brain injury on neuroimaging during the intensive care unit (ICU) stay, (c) new central nervous system disorders with clinical manifestation, and (d) functional outcome at three months. ^b^Other findings include hygroma (n=2), right and left parietal lesions indicative for cerebral aspergillosis (n=1), global brain edema (n=1).
