# Supplementary material for: Cerebrovascular autoregulation and arterial carbon dioxide in patients with acute respiratory distress syndrome: a prospective observational cohort study
Source: Ann Intensive Care. 2021 Mar 16;11:47. doi: 10.1186/s13613-021-00831-7 (PMC7962086; doi:10.1186/s13613-021-00831-7)
Supplement: Supplementary file 4 — Additional file 4. Development of arterial carbon dioxide. PaCO2 from the diagnosis of ARDS until first CVA assessment and between first and second CVA assessments, stratified by the presence of early hypercapnia. [file 13613_2021_831_MOESM4_ESM.docx]

**Additional file 4**

**Additional file 4a**: PaCO_2_ between the diagnosis of ARDS and the first CVA measurement in patients with and without early hypercapnia. The total number of arterial blood gas analyses differed between groups.

**Additional file 4b**: PaCO_2_ between the first and the second CVA measurement in patients with and without early hypercapnia. The total number of arterial blood gas analyses differed between groups.
