# Supplementary material for: Cerebrovascular autoregulation and arterial carbon dioxide in patients with acute respiratory distress syndrome: a prospective observational cohort study
Source: Ann Intensive Care. 2021 Mar 16;11:47. doi: 10.1186/s13613-021-00831-7 (PMC7962086; doi:10.1186/s13613-021-00831-7)
Supplement: Supplementary file 5 — Additional file 5. Linear mixed model including preselected variables of clinical relevance. Initial linear mixed model. All variables included were selected based on clinical considerations. [file 13613_2021_831_MOESM5_ESM.docx]

**Additional file 5**

|  | Estimate | 95% CI lower limit | 95% CI upper limit | *p* |
| --- | --- | --- | --- | --- |
| *Intercept* | 0.124 | -0.173 | 0.420 | 0.409 |
| *No early hypercapnia (reference: early hypercapnia PaCO_2_ ≥50 mmHg)* | 0.021 | -0.059 | 0.102 | 0.592 |
| *vv-ECMO at the time of CVA measurement (reference: no vv-ECMO)* | 0.024 | -0.100 | 0.148 | 0.701 |
| *Supine position (reference: prone position)* | 0.014 | -0.078 | 0.105 | 0.767 |
| *Sedation (reference: no sedation)* |  |  |  |  |
| inhalational | -0.040 | -0.225 | 0.146 | 0.671 |
| intranveous | -0.069 | -0.242 | 0.103 | 0.427 |
| mixed | -0.069 | -0.266 | 0.127 | 0.487 |
| *ARDS severity (reference: severe)* |  |  |  |  |
| mild | 0.016 | -0.111 | 0.143 | 0.801 |
| moderate | -0.032 | -0.140 | 0.076 | 0.556 |
| *No inhalational nitric oxide (Reference: inhalational nitric oxide at time of measurement)* | 0.026 | -0.073 | 0.125 | 0.603 |
| *Age (per year increase)* | 0.001 | -0.001 | 0.004 | 0.343 |
| *SOFA score (per point increase)* | 0.002 | -0.010 | 0.014 | 0.793 |
| *Hypocapnia at time of measurement (reference: normo- and hypercapnia)* | 0.153 | 0.009 | 0.297 | 0.037 |
| *Community-acquired ARDS (reference: hospital-acquired ARDS)* | 0.050 | -0.030 | 0.130 | 0.217 |

**Additional file 5**: Linear mixed model – estimates of fixed effects. The initial model included variables that were considered clinically relevant. PaCO_2_: arterial partial pressure of carbon dioxide. Vv-ECMO: veno-venous extracorporeal membrane oxygenation. ARDS: acute respiratory distress syndrome. SOFA: Sequential Organ Failure Assessment.
