# Supplementary material for: Cerebrovascular autoregulation and arterial carbon dioxide in patients with acute respiratory distress syndrome: a prospective observational cohort study
Source: Ann Intensive Care. 2021 Mar 16;11:47. doi: 10.1186/s13613-021-00831-7 (PMC7962086; doi:10.1186/s13613-021-00831-7)
Supplement: Supplementary file 6 — Additional file 6. Sensitivity analysis. 6a—Linear mixed model with an alternative definition of early hypercapnia (PaCO2 ≥ 60 mmHg with pH < 7.35). 6b—Linear mixed model with delta PaCO2 (difference between ARDS diagnosis and CVA measurements) as independent variable. [file 13613_2021_831_MOESM6_ESM.docx]

**Additional file 6**

|  | **Estimate** | **95% CI - Low** | **95% CI - Up** | ***p*** |
| --- | --- | --- | --- | --- |
| ***Intercept*** | 0.117 | -0.020 | 0.255 | 0.093 |
| ***No early hypercapnia (vs. early hypercapnia = PaCO_2_ ≥60 mmHg)*** | 0.045 | -0.024 | 0.114 | 0.196 |
| ***Age (per year increase)*** | 0.001 | -0.001 | 0.003 | 0.352 |
| ***Hypocapnia during the measurement period^a^*** | 0.186 | 0.072 | 0.300 | 0.002 |
| ***ARDS etiology (community-acquired vs. hospital-acquired)^b^*** | 0.049 | -0.021 | 0.120 | 0.166 |

Additional file 6a. Sensitivity analysis with a different definition of early hypercapnia (PaCO_2_ ≥ 60 mmHg and pH <7.35). Linear mixed model - estimates of fixed effects. The variables ARDS severity (mild / moderate / severe) and sedation (none / intravenous / inhalational / mixed), position (prone vs. supine), inhaled nitric oxide, extracorporeal membrane oxygenation, and the Sequential Organ Failure Assessment score during measurement were included in the initial model and eliminated during the stepwise-backwards reduction. ARDS: acute respiratory distress syndrome. ^a^Vs. Normo- and hypercapnia. ^b^Etiologies were categorized as “community-acquired” and “hospital-acquired” for the linear mixed model.

|  | **Estimate** | **95% CI - Low** | **95% CI - Up** | ***p*** |
| --- | --- | --- | --- | --- |
| ***Intercept*** | 0.1921 | 0.0497 | 0.3344 | 0.009 |
| ***Delta PaCO_2_ (per mmHg increase)^a^*** | -0.0004 | -0.0018 | 0.0009 | 0.507 |
| ***No ECMO (vs. ECMO during measurement)^b^*** | -0.0125 | -0.1059 | 0.0809 | 0.791 |
| ***Age (per year increase)*** | 0.0010 | -0.0014 | 0.0033 | 0.403 |
| ***Hypocapnia during the measurement period^c^*** | 0.1815 | 0.0664 | 0.2967 | 0.002 |
| Additional file bb: Sensitivity analysis with delta PaCO_2_. Linear mixed model - estimates of fixed effects. The variables position (prone vs. supine), sedation, ARDS severity, inhaled nitric oxide, ARDS etiology (community-acquired vs. hospital-acquired) and the Sequential Organ Failure Assessment score during measurement were included in the initial model and eliminated during the stepwise-backwards reduction-process. ARDS: acute respiratory distress syndrome. ^a^Difference in PaCO_2_ between ARDS diagnosis and CVA assessment. ^b^Extracorporeal membrane oxygenation. ^c^Vs. Normo- and hypercapnia | | | | |
