# Supplementary material for: Cerebrovascular autoregulation and arterial carbon dioxide in patients with acute respiratory distress syndrome: a prospective observational cohort study
Source: Ann Intensive Care. 2021 Mar 16;11:47. doi: 10.1186/s13613-021-00831-7 (PMC7962086; doi:10.1186/s13613-021-00831-7)
Supplement: Supplementary file 7 — Additional file 7. CVA in patients with and without ECMO. 7a—Hemodynamic parameters and selected results from blood gas analyses in patients requiring extracorporeal membrane oxygenation (ECMO) and patients without ECMO. Mean values from monitoring episodes 1 and 2 are presented, stratified by the requirement of veno-venous ECMO. Data are given as mean ± SD. COx: cerebral oxygenation index representing cerebrovascular autoregulation (CVA). rSO2: cerebral oxygenation measured with near-infrared spectroscopy. MAP: mean arterial blood pressure. 7b—Time with impaired CVA between 19 patients with veno-venous ECMO, and 47 patients without ECMO. [file 13613_2021_831_MOESM7_ESM.docx]

**Additional file 7**

|  | No ECMO  (n=47) | ECMO  (n=19) |
| --- | --- | --- |
|  | *Measurement*  *periods=86* | *Measurement*  *periods=31* |
| Time with impaired CVA (%) | 25.84 ± 17.94 | 21.76 ± 20.7 |
| Cerebral oxygenation index COx | 0.1 ± 0.14 | 0.01 ± 0.22 |
| rSO_2_ (%) | 63.21 ± 11.6 | 67.32 ± 10.96 |
| MAP (mmHg) | 75.53 ± 10.27 | 75.23 ± 8.66 |
| Haemoglobin (mg*dl^-1^) | 9.4 ± 1.7 | 10 ± 0.9 |
| Lactate (mmol/l) | 1.8 ± 1.5 | 2.6 ± 3 |
| PaO_2_/FiO_2_ ratio | 176.8 ± 62.5 | 82.6 ± 15.7 |
| PaCO_2_ (mmHg) | 47.1 ± 11.3 | 45.7 ± 6.3 |
| pH | 7.4 ± 0.08 | 7.41 ± 0.1 |
| Δ^a^ FiO_2_/PaO_2_ ratio | 10.5 ± 19.5 | 5.5 ± 7.3 |
| Δ^a^ PaCO_2_ (mmHg) | 1.36 ± 2.64 | 2.2 ± 2.9 |
| Δ^a^ pH | 0.01 ± 0.02 | 0.01 ± 0.03 |
| Additional file 7a: Hemodynamic parameters and selected results from blood gas analyses in patients requiring extracorporeal membrane oxygenation (ECMO) and patients without ECMO. Mean values from monitoring episodes 1 and 2 are presented, stratified by the requirement of veno-venous ECMO. Data are given as mean ± SD. COx: cerebral oxygenation index representing cerebrovascular autoregulation (CVA). rSO_2_: cerebral oxygenation measured with near-infrared spectroscopy. MAP: mean arterial blood pressure. ^a^Δvalues refer to the mean difference between the first and the second CVA measurement in one study participant. | | |

**Additional file 7b**: Time with impaired cerebrovascular autoregulation (CVA) between 19 patients with veno-venous extracorporeal membrane oxygenation (ECMO) and 47 patients without ECMO.
