# Supplementary material for: Cerebrovascular autoregulation and arterial carbon dioxide in patients with acute respiratory distress syndrome: a prospective observational cohort study
Source: Ann Intensive Care. 2021 Mar 16;11:47. doi: 10.1186/s13613-021-00831-7 (PMC7962086; doi:10.1186/s13613-021-00831-7)
Supplement: Supplementary file 8 — Additional file 8. Repeated CVA measurements in 7 patients with and without ECMO. 8a—Repeated measurement episodes of cerebrovascular autoregulation (CVA) with and without extracorporeal membrane oxygenation (ECMO) in 7 patients. Data are given as mean ± SD. COx: cerebral oxygenation index as a surrogate of CVA. rSO2: cerebral oxygenation measured with near-infrared spectroscopy. 8b—Time with impaired CVA in repeated measurements with and without ECMO. [file 13613_2021_831_MOESM8_ESM.docx]

**Additional file 8**

|  | No ECMO  (n=7) | ECMO  (n=7) |
| --- | --- | --- |
| Time with impaired CVA (%) | 31.91 ± 16.27 | 29.93 ± 31.90 |
| Cerebral oxygenation index COx | 0.14 ± 0.13 | 0.14 ± 0.33 |
| rSO_2_ (%) | 68.23 ± 9.67 | 61.12 ± 10.36 |
| MAP (mmHg) | 74.64 ± 10.27 | 78.56 ± 9.17 |
| Haemoglobin (mg*dl^-1^) | 9.8 ± 1.3 | 9.6 ± 0.6 |
| Lactate (mmol/l) | 2.7 ± 3 | 4.1 ± 6 |
| PaO_2_/FiO_2_ ratio | 170.4 ± 73.4 | 92.4 ± 12.5 |
| PaCO_2_ (mmHg) | 51.7 ± 16.6 | 42.6 ± 5.8 |
| pH | 7.36 ± 0.1 | 7.38 ± 0.18 |
| Δ^a^ FiO_2_/PaO_2_ ratio | 7.6 ± 10.0 | 7.9 ± 8.1 |
| Δ^a^ PaCO_2_ (mmHg) | 1.0 ± 1.0 | 2.5 ± 3.4 |
| Δ^a^ pH | 0.01 ± 0.02 | 0.02 ± 0.03 |
| Additional file 8a: Repeated measurement episodes of cerebrovascular autoregulation (CVA) with and without extracorporeal membrane oxygenation (ECMO) in 7 patients. Data are given as mean ± SD. COx: cerebral oxygenation index as a surrogate of CVA. rSO_2_: cerebral oxygenation measured with near-infrared spectroscopy. ^a^Δvalues refer to the mean difference between the first and the second CVA measurement in one study participant. | | |

**Additional file 8b**: Time with impaired cerebrovascular autoregulation (CVA) in paired monitoring episodes of 7 patients during veno-venous extracorporeal membrane oxygenation (ECMO) and without ECMO.
